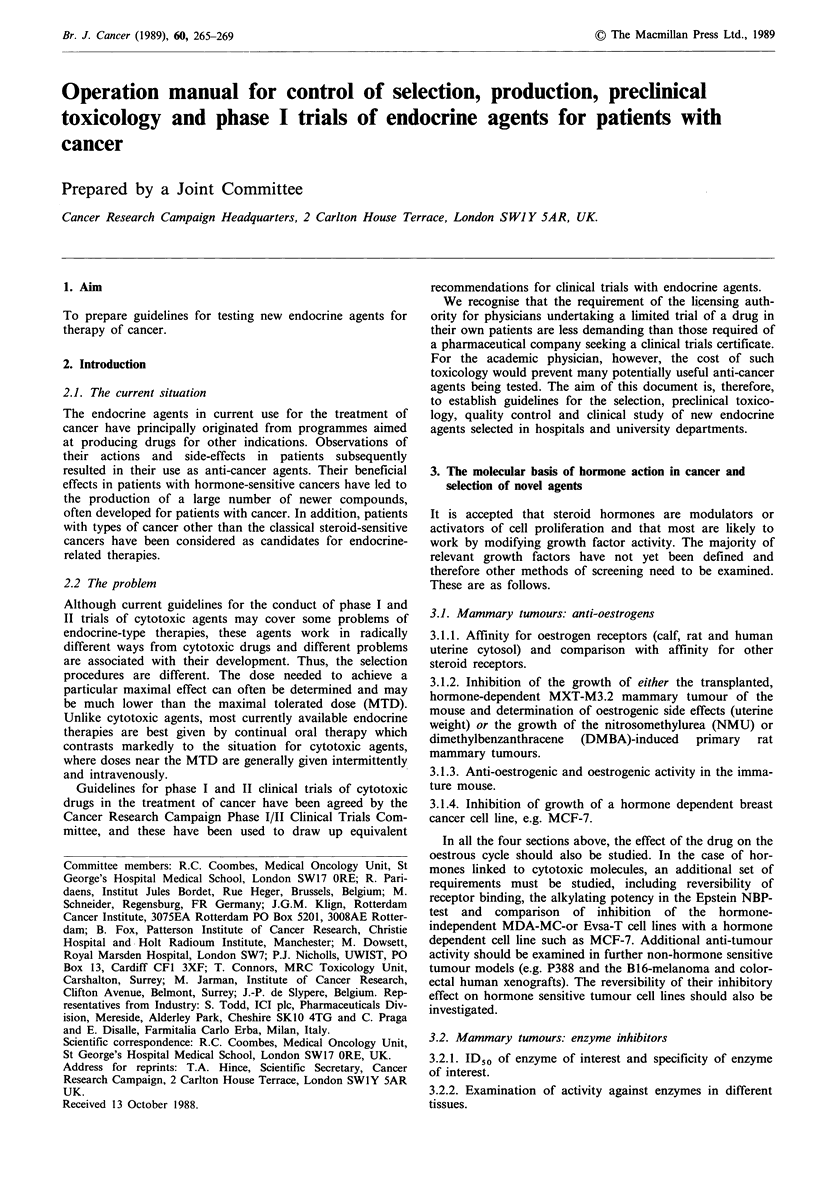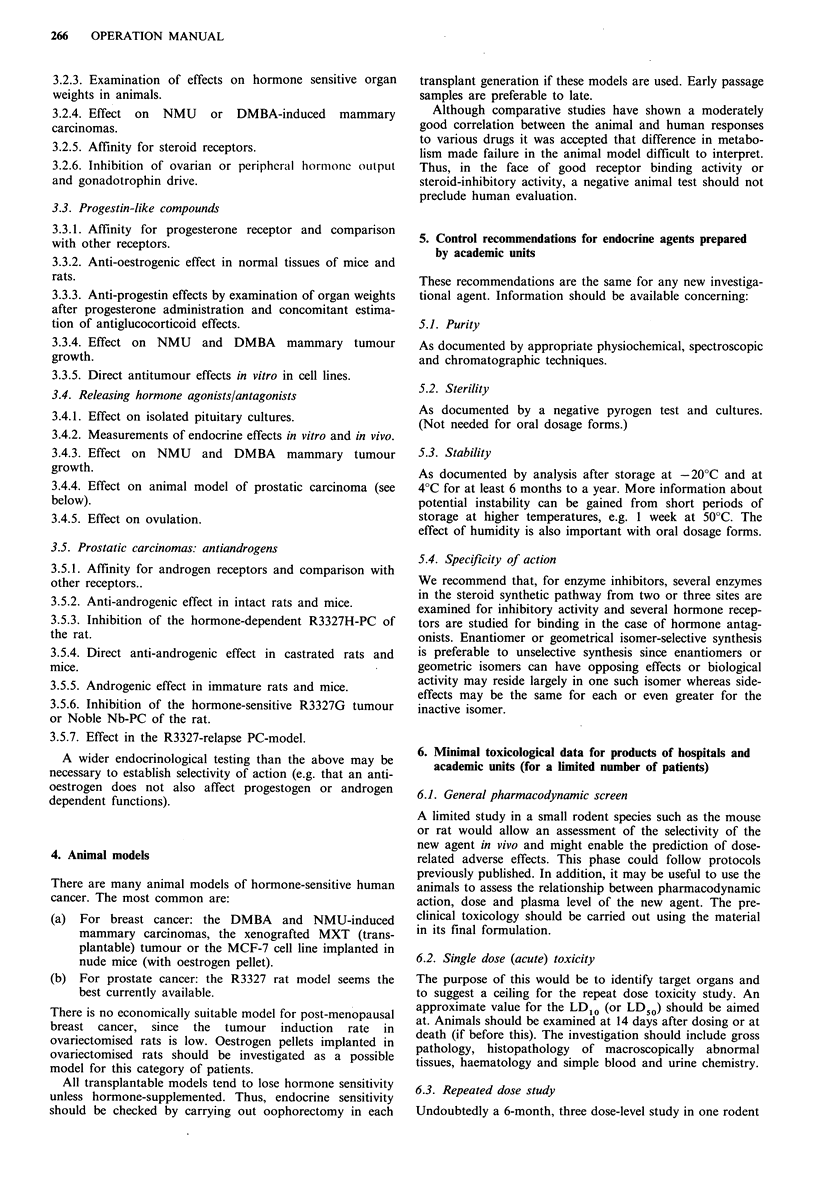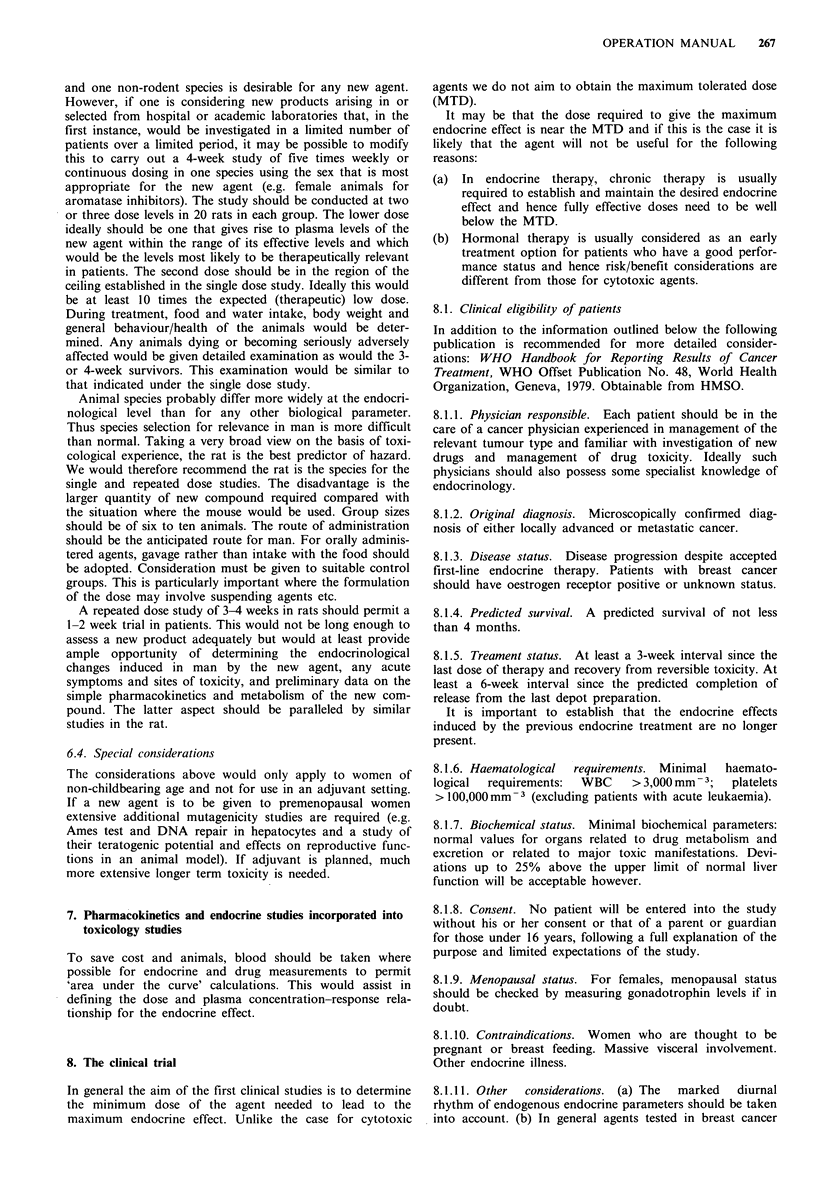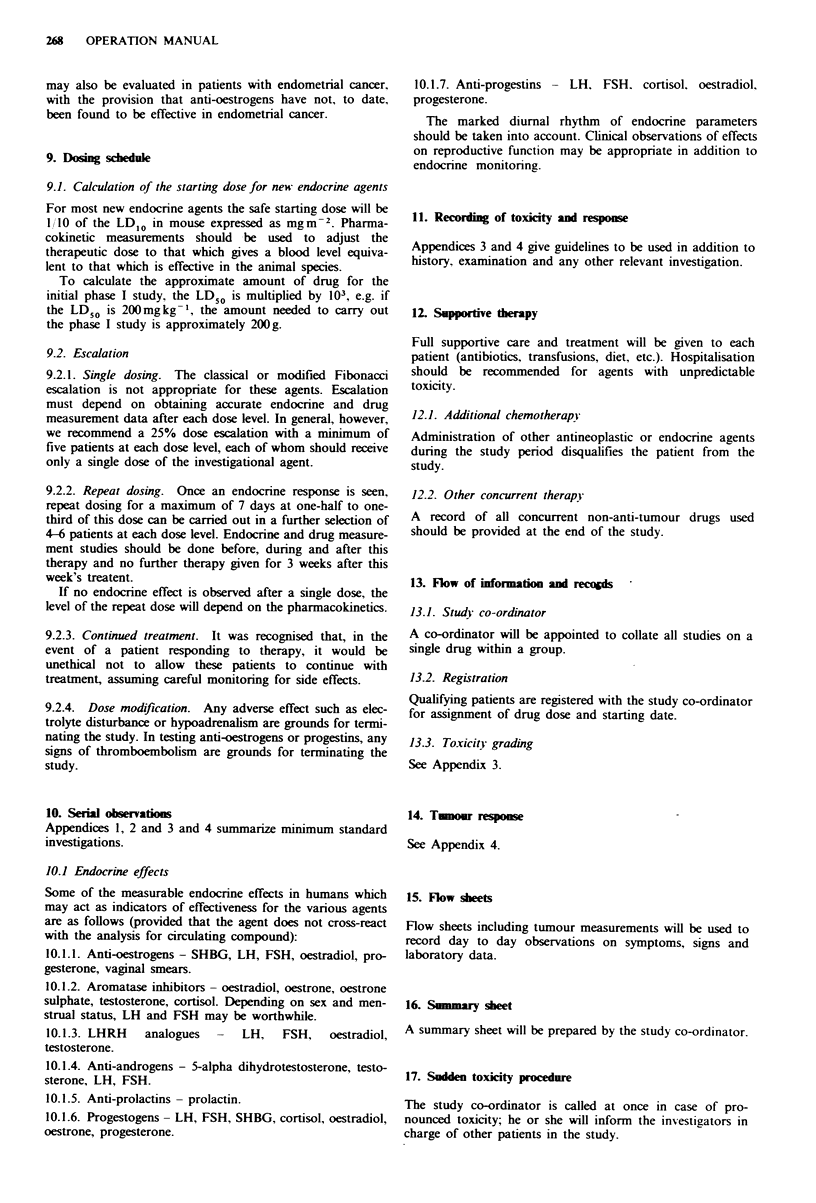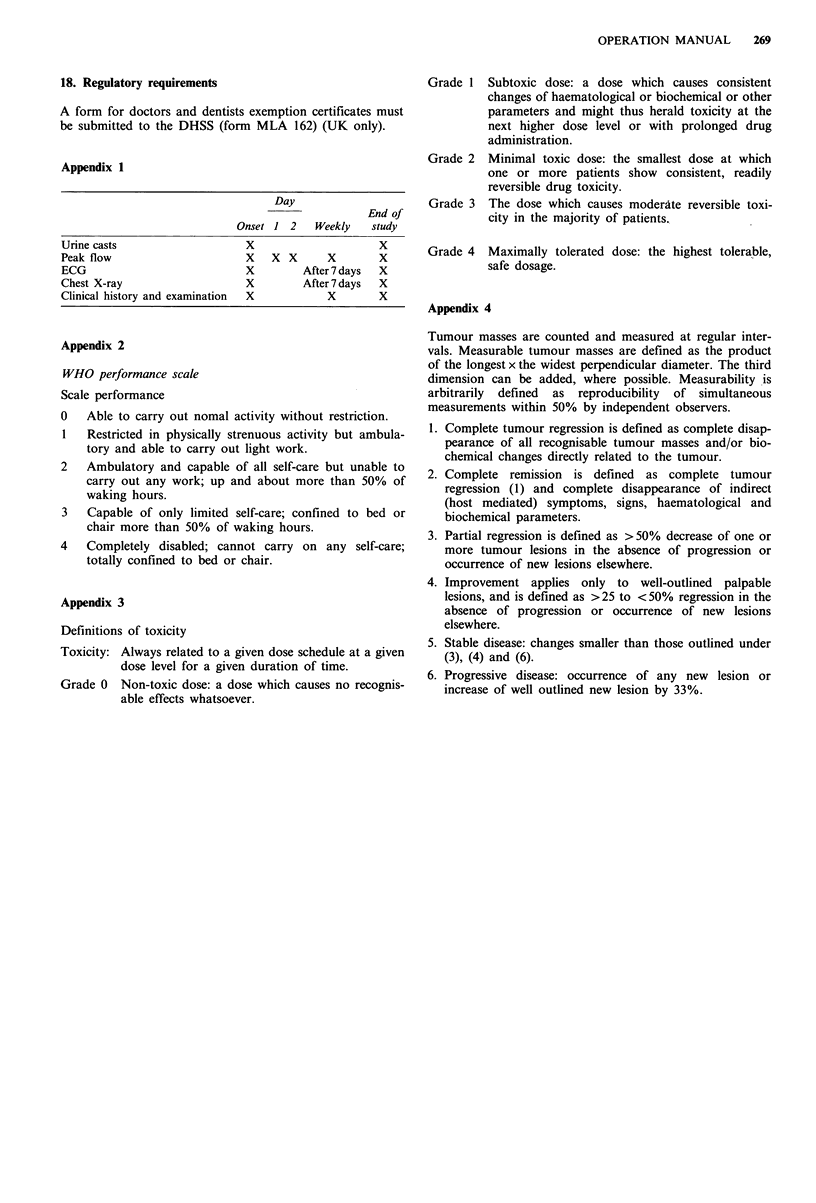# Operation manual for control of selection, production, preclinical toxicology and phase I trials of endocrine agents for patients with cancer.

**DOI:** 10.1038/bjc.1989.267

**Published:** 1989-08

**Authors:** 


*Br. J. Cancer* (1989), **60**, 265–269 © The Macmillan Press Ltd., 1989

### Operation manual for control of selection, production, preclinical toxicology and phase I trials of endocrine agents for patients with cancer

Prepared by a Joint Committee

*Cancer Research Campaign Headquarters, 2 Carlton House Terrace, London SW1Y 5AR, UK.*

### 1. Aim

To prepare guidelines for testing new endocrine agents for therapy of cancer.

### 2. Introduction

### *2.1. The current situation*

The endocrine agents in current use for the treatment of cancer have principally originated from programmes aimed at producing drugs for other indications. Observations of their actions and side-effects in patients subsequently resulted in their use as anti-cancer agents. Their beneficial effects in patients with hormone-sensitive cancers have led to the production of a large number of newer compounds, often developed for patients with cancer. In addition, patients with types of cancer other than the classical steroid-sensitive cancers have been considered as candidates for endocrine-related therapies.

### *2.2 The problem*

Although current guidelines for the conduct of phase I and II trials of cytotoxic agents may cover some problems of endocrine-type therapies, these agents work in radically different ways from cytotoxic drugs and different problems are associated with their development. Thus, the selection procedures are different. The dose needed to achieve a particular maximal effect can often be determined and may be much lower than the maximal tolerated dose (MTD). Unlike cytotoxic agents, most currently available endocrine therapies are best given by continual oral therapy which contrasts markedly to the situation for cytotoxic agents, where doses near the MTD are generally given intermittently and intravenously.

Guidelines for phase I and II clinical trials of cytotoxic drugs in the treatment of cancer have been agreed by the Cancer Research Campaign Phase I/II Clinical Trials Committee, and these have been used to draw up equivalent recommendations for clinical trials with endocrine agents.

We recognise that the requirement of the licensing authority for physicians undertaking a limited trial of a drug in their own patients are less demanding than those required of a pharmaceutical company seeking a clinical trials certificate. For the academic physician, however, the cost of such toxicology would prevent many potentially useful anti-cancer agents being tested. The aim of this document is, therefore, to establish guidelines for the selection, preclinical toxicology, quality control and clinical study of new endocrine agents selected in hospitals and university departments.

### 3. The molecular basis of hormone action in cancer and selection of novel agents

It is accepted that steroid hormones are modulators or activators of cell proliferation and that most are likely to work by modifying growth factor activity. The majority of relevant growth factors have not yet been defined and therefore other methods of screening need to be examined. These are as follows.

### *3.1. Mammary tumours: anti-oestrogens*

3.1.1. Affinity for oestrogen receptors (calf, rat and human uterine cytosol) and comparison with affinity for other steroid receptors.

3.1.2. Inhibition of the growth of *either* the transplanted, hormone-dependent MXT-M3.2 mammary tumour of the mouse and determination of oestrogenic side effects (uterine weight) *or* the growth of the nitrosomethylurea (NMU) or dimethylbenzanthracene (DMBA)-induced primary rat mammary tumours.

3.1.3. Anti-oestrogenic and oestrogenic activity in the immature mouse.

3.1.4. Inhibition of growth of a hormone dependent breast cancer cell line, e.g. MCF-7.

In all the four sections above, the effect of the drug on the oestrous cycle should also be studied. In the case of hormones linked to cytotoxic molecules, an additional set of requirements must be studied, including reversibility of receptor binding, the alkylating potency in the Epstein NBP-test and comparison of inhibition of the hormone-independent MDA-MC-or Evsa-T cell lines with a hormone dependent cell line such as MCF-7. Additional anti-tumour activity should be examined in further non-hormone sensitive tumour models (e.g. P388 and the B16-melanoma and colorectal human xenografts). The reversibility of their inhibitory effect on hormone sensitive tumour cell lines should also be investigated.

### *3.2. Mammary tumours: enzyme inhibitors*

3.2.1. $ID_{50}$ of enzyme of interest and specificity of enzyme of interest.

3.2.2. Examination of activity against enzymes in different tissues.

Committee members: R.C. Coombes, Medical Oncology Unit, St George's Hospital Medical School, London SW17 0RE; R. Paridaens, Institut Jules Bordet, Rue Heger, Brussels, Belgium; M. Schneider, Regensburg, FR Germany; J.G.M. Klign, Rotterdam Cancer Institute, 3075EA Rotterdam PO Box 5201, 3008AE Rotterdam; B. Fox, Patterson Institute of Cancer Research, Christie Hospital and Holt Radioum Institute, Manchester; M. Dowsett, Royal Marsden Hospital, London SW7; P.J. Nicholls, UWIST, PO Box 13, Cardiff CF1 3XF; T. Connors, MRC Toxicology Unit, Carshalton, Surrey; M. Jarman, Institute of Cancer Research, Clifton Avenue, Belmont, Surrey; J.-P. de Slypere, Belgium. Representatives from Industry: S. Todd, ICI plc, Pharmaceuticals Division, Mereside, Alderley Park, Cheshire SK10 4TG and C. Praga and E. Disalle, Farmitalia Carlo Erba, Milan, Italy.
Scientific correspondence: R.C. Coombes, Medical Oncology Unit, St George's Hospital Medical School, London SW17 0RE, UK.
Address for reprints: T.A. Hince, Scientific Secretary, Cancer Research Campaign, 2 Carlton House Terrace, London SW1Y 5AR UK.
Received 13 October 1988.

3.2.3. Examination of effects on hormone sensitive organ weights in animals.

3.2.4. Effect on NMU or DMBA-induced mammary carcinomas.

3.2.5. Affinity for steroid receptors.

3.2.6. Inhibition of ovarian or peripheral hormone output and gonadotrophin drive.

*3.3. Progestin-like compounds*

3.3.1. Affinity for progesterone receptor and comparison with other receptors.

3.3.2. Anti-oestrogenic effect in normal tissues of mice and rats.

3.3.3. Anti-progestin effects by examination of organ weights after progesterone administration and concomitant estimation of antiglucocorticoid effects.

3.3.4. Effect on NMU and DMBA mammary tumour growth.

3.3.5. Direct antitumour effects *in vitro* in cell lines.

*3.4. Releasing hormone agonists/antagonists*

3.4.1. Effect on isolated pituitary cultures.

3.4.2. Measurements of endocrine effects *in vitro* and *in vivo*.

3.4.3. Effect on NMU and DMBA mammary tumour growth.

3.4.4. Effect on animal model of prostatic carcinoma (see below).

3.4.5. Effect on ovulation.

*3.5. Prostatic carcinomas: antiandrogens*

3.5.1. Affinity for androgen receptors and comparison with other receptors..

3.5.2. Anti-androgenic effect in intact rats and mice.

3.5.3. Inhibition of the hormone-dependent R3327H-PC of the rat.

3.5.4. Direct anti-androgenic effect in castrated rats and mice.

3.5.5. Androgenic effect in immature rats and mice.

3.5.6. Inhibition of the hormone-sensitive R3327G tumour or Noble Nb-PC of the rat.

3.5.7. Effect in the R3327-relapse PC-model.

A wider endocrinological testing than the above may be necessary to establish selectivity of action (e.g. that an anti-oestrogen does not also affect progestogen or androgen dependent functions).

### 4. Animal models

There are many animal models of hormone-sensitive human cancer. The most common are:

(a) For breast cancer: the DMBA and NMU-induced mammary carcinomas, the xenografted MXT (transplantable) tumour or the MCF-7 cell line implanted in nude mice (with oestrogen pellet).

(b) For prostate cancer: the R3327 rat model seems the best currently available.

There is no economically suitable model for post-menopausal breast cancer, since the tumour induction rate in ovariectomised rats is low. Oestrogen pellets implanted in ovariectomised rats should be investigated as a possible model for this category of patients.

All transplantable models tend to lose hormone sensitivity unless hormone-supplemented. Thus, endocrine sensitivity should be checked by carrying out oophorectomy in each transplant generation if these models are used. Early passage samples are preferable to late.

Although comparative studies have shown a moderately good correlation between the animal and human responses to various drugs it was accepted that difference in metabolism made failure in the animal model difficult to interpret. Thus, in the face of good receptor binding activity or steroid-inhibitory activity, a negative animal test should not preclude human evaluation.

### 5. Control recommendations for endocrine agents prepared by academic units

These recommendations are the same for any new investigational agent. Information should be available concerning:

*5.1. Purity*

As documented by appropriate physiochemical, spectroscopic and chromatographic techniques.

*5.2. Sterility*

As documented by a negative pyrogen test and cultures. (Not needed for oral dosage forms.)

*5.3. Stability*

As documented by analysis after storage at −20°C and at 4°C for at least 6 months to a year. More information about potential instability can be gained from short periods of storage at higher temperatures, e.g. 1 week at 50°C. The effect of humidity is also important with oral dosage forms.

*5.4. Specificity of action*

We recommend that, for enzyme inhibitors, several enzymes in the steroid synthetic pathway from two or three sites are examined for inhibitory activity and several hormone receptors are studied for binding in the case of hormone antagonists. Enantiomer or geometrical isomer-selective synthesis is preferable to unselective synthesis since enantiomers or geometric isomers can have opposing effects or biological activity may reside largely in one such isomer whereas side-effects may be the same for each or even greater for the inactive isomer.

### 6. Minimal toxicological data for products of hospitals and academic units (for a limited number of patients)

*6.1. General pharmacodynamic screen*

A limited study in a small rodent species such as the mouse or rat would allow an assessment of the selectivity of the new agent *in vivo* and might enable the prediction of dose-related adverse effects. This phase could follow protocols previously published. In addition, it may be useful to use the animals to assess the relationship between pharmacodynamic action, dose and plasma level of the new agent. The pre-clinical toxicology should be carried out using the material in its final formulation.

*6.2. Single dose (acute) toxicity*

The purpose of this would be to identify target organs and to suggest a ceiling for the repeat dose toxicity study. An approximate value for the $LD_{10}$ (or $LD_{50}$) should be aimed at. Animals should be examined at 14 days after dosing or at death (if before this). The investigation should include gross pathology, histopathology of macroscopically abnormal tissues, haematology and simple blood and urine chemistry.

*6.3. Repeated dose study*

Undoubtedly a 6-month, three dose-level study in one rodent and one non-rodent species is desirable for any new agent. However, if one is considering new products arising in or selected from hospital or academic laboratories that, in the first instance, would be investigated in a limited number of patients over a limited period, it may be possible to modify this to carry out a 4-week study of five times weekly or continuous dosing in one species using the sex that is most appropriate for the new agent (e.g. female animals for aromatase inhibitors). The study should be conducted at two or three dose levels in 20 rats in each group. The lower dose ideally should be one that gives rise to plasma levels of the new agent within the range of its effective levels and which would be the levels most likely to be therapeutically relevant in patients. The second dose should be in the region of the ceiling established in the single dose study. Ideally this would be at least 10 times the expected (therapeutic) low dose. During treatment, food and water intake, body weight and general behaviour/health of the animals would be determined. Any animals dying or becoming seriously adversely affected would be given detailed examination as would the 3- or 4-week survivors. This examination would be similar to that indicated under the single dose study.

Animal species probably differ more widely at the endocrinological level than for any other biological parameter. Thus species selection for relevance in man is more difficult than normal. Taking a very broad view on the basis of toxicological experience, the rat is the best predictor of hazard. We would therefore recommend the rat is the species for the single and repeated dose studies. The disadvantage is the larger quantity of new compound required compared with the situation where the mouse would be used. Group sizes should be of six to ten animals. The route of administration should be the anticipated route for man. For orally administered agents, gavage rather than intake with the food should be adopted. Consideration must be given to suitable control groups. This is particularly important where the formulation of the dose may involve suspending agents etc.

A repeated dose study of 3–4 weeks in rats should permit a 1–2 week trial in patients. This would not be long enough to assess a new product adequately but would at least provide ample opportunity of determining the endocrinological changes induced in man by the new agent, any acute symptoms and sites of toxicity, and preliminary data on the simple pharmacokinetics and metabolism of the new compound. The latter aspect should be paralleled by similar studies in the rat.

### *6.4. Special considerations*

The considerations above would only apply to women of non-childbearing age and not for use in an adjuvant setting. If a new agent is to be given to premenopausal women extensive additional mutagenicity studies are required (e.g. Ames test and DNA repair in hepatocytes and a study of their teratogenic potential and effects on reproductive functions in an animal model). If adjuvant is planned, much more extensive longer term toxicity is needed.

### 7. Pharmacokinetics and endocrine studies incorporated into toxicology studies

To save cost and animals, blood should be taken where possible for endocrine and drug measurements to permit 'area under the curve' calculations. This would assist in defining the dose and plasma concentration–response relationship for the endocrine effect.

### 8. The clinical trial

In general the aim of the first clinical studies is to determine the minimum dose of the agent needed to lead to the maximum endocrine effect. Unlike the case for cytotoxic agents we do not aim to obtain the maximum tolerated dose (MTD).

It may be that the dose required to give the maximum endocrine effect is near the MTD and if this is the case it is likely that the agent will not be useful for the following reasons:

(a) In endocrine therapy, chronic therapy is usually required to establish and maintain the desired endocrine effect and hence fully effective doses need to be well below the MTD.

(b) Hormonal therapy is usually considered as an early treatment option for patients who have a good performance status and hence risk/benefit considerations are different from those for cytotoxic agents.

### 8.1. *Clinical eligibility of patients*

In addition to the information outlined below the following publication is recommended for more detailed considerations: *WHO Handbook for Reporting Results of Cancer Treatment,* WHO Offset Publication No. 48, World Health Organization, Geneva, 1979. Obtainable from HMSO.

8.1.1. *Physician responsible.* Each patient should be in the care of a cancer physician experienced in management of the relevant tumour type and familiar with investigation of new drugs and management of drug toxicity. Ideally such physicians should also possess some specialist knowledge of endocrinology.

8.1.2. *Original diagnosis.* Microscopically confirmed diagnosis of either locally advanced or metastatic cancer.

8.1.3. *Disease status.* Disease progression despite accepted first-line endocrine therapy. Patients with breast cancer should have oestrogen receptor positive or unknown status.

8.1.4. *Predicted survival.* A predicted survival of not less than 4 months.

8.1.5. *Treament status.* At least a 3-week interval since the last dose of therapy and recovery from reversible toxicity. At least a 6-week interval since the predicted completion of release from the last depot preparation.

It is important to establish that the endocrine effects induced by the previous endocrine treatment are no longer present.

8.1.6. *Haematological requirements.* Minimal haematological requirements: WBC $>3{,}000\,mm^{-3}$; platelets $>100{,}000\,mm^{-3}$ (excluding patients with acute leukaemia).

8.1.7. *Biochemical status.* Minimal biochemical parameters: normal values for organs related to drug metabolism and excretion or related to major toxic manifestations. Deviations up to 25% above the upper limit of normal liver function will be acceptable however.

8.1.8. *Consent.* No patient will be entered into the study without his or her consent or that of a parent or guardian for those under 16 years, following a full explanation of the purpose and limited expectations of the study.

8.1.9. *Menopausal status.* For females, menopausal status should be checked by measuring gonadotrophin levels if in doubt.

8.1.10. *Contraindications.* Women who are thought to be pregnant or breast feeding. Massive visceral involvement. Other endocrine illness.

8.1.11. *Other considerations.* (a) The marked diurnal rhythm of endogenous endocrine parameters should be taken into account. (b) In general agents tested in breast cancer may also be evaluated in patients with endometrial cancer, with the provision that anti-oestrogens have not, to date, been found to be effective in endometrial cancer.

### 9. Dosing schedule

*9.1. Calculation of the starting dose for new endocrine agents*

For most new endocrine agents the safe starting dose will be 1/10 of the $LD_{10}$ in mouse expressed as $mg\,m^{-2}$. Pharmacokinetic measurements should be used to adjust the therapeutic dose to that which gives a blood level equivalent to that which is effective in the animal species.

To calculate the approximate amount of drug for the initial phase I study, the $LD_{50}$ is multiplied by $10^3$, e.g. if the $LD_{50}$ is $200\,mg\,kg^{-1}$, the amount needed to carry out the phase I study is approximately 200 g.

*9.2. Escalation*

9.2.1. *Single dosing.* The classical or modified Fibonacci escalation is not appropriate for these agents. Escalation must depend on obtaining accurate endocrine and drug measurement data after each dose level. In general, however, we recommend a 25% dose escalation with a minimum of five patients at each dose level, each of whom should receive only a single dose of the investigational agent.

9.2.2. *Repeat dosing.* Once an endocrine response is seen, repeat dosing for a maximum of 7 days at one-half to one-third of this dose can be carried out in a further selection of 4–6 patients at each dose level. Endocrine and drug measurement studies should be done before, during and after this therapy and no further therapy given for 3 weeks after this week's treatent.

If no endocrine effect is observed after a single dose, the level of the repeat dose will depend on the pharmacokinetics.

9.2.3. *Continued treatment.* It was recognised that, in the event of a patient responding to therapy, it would be unethical not to allow these patients to continue with treatment, assuming careful monitoring for side effects.

9.2.4. *Dose modification.* Any adverse effect such as electrolyte disturbance or hypoadrenalism are grounds for terminating the study. In testing anti-oestrogens or progestins, any signs of thromboembolism are grounds for terminating the study.

### 10. Serial observations

Appendices 1, 2 and 3 and 4 summarize minimum standard investigations.

*10.1 Endocrine effects*

Some of the measurable endocrine effects in humans which may act as indicators of effectiveness for the various agents are as follows (provided that the agent does not cross-react with the analysis for circulating compound):

10.1.1. Anti-oestrogens – SHBG, LH, FSH, oestradiol, progesterone, vaginal smears.

10.1.2. Aromatase inhibitors – oestradiol, oestrone, oestrone sulphate, testosterone, cortisol. Depending on sex and menstrual status, LH and FSH may be worthwhile.

10.1.3. LHRH analogues – LH, FSH, oestradiol, testosterone.

10.1.4. Anti-androgens – 5-alpha dihydrotestosterone, testosterone, LH, FSH.

10.1.5. Anti-prolactins – prolactin.

10.1.6. Progestogens – LH, FSH, SHBG, cortisol, oestradiol, oestrone, progesterone.

10.1.7. Anti-progestins – LH, FSH, cortisol, oestradiol, progesterone.

The marked diurnal rhythm of endocrine parameters should be taken into account. Clinical observations of effects on reproductive function may be appropriate in addition to endocrine monitoring.

### 11. Recording of toxicity and response

Appendices 3 and 4 give guidelines to be used in addition to history, examination and any other relevant investigation.

### 12. Supportive therapy

Full supportive care and treatment will be given to each patient (antibiotics, transfusions, diet, etc.). Hospitalisation should be recommended for agents with unpredictable toxicity.

*12.1. Additional chemotherapy*

Administration of other antineoplastic or endocrine agents during the study period disqualifies the patient from the study.

*12.2. Other concurrent therapy*

A record of all concurrent non-anti-tumour drugs used should be provided at the end of the study.

### 13. Flow of information and records

*13.1. Study co-ordinator*

A co-ordinator will be appointed to collate all studies on a single drug within a group.

*13.2. Registration*

Qualifying patients are registered with the study co-ordinator for assignment of drug dose and starting date.

*13.3. Toxicity grading*

See Appendix 3.

### 14. Tumour response

See Appendix 4.

### 15. Flow sheets

Flow sheets including tumour measurements will be used to record day to day observations on symptoms, signs and laboratory data.

### 16. Summary sheet

A summary sheet will be prepared by the study co-ordinator.

### 17. Sudden toxicity procedure

The study co-ordinator is called at once in case of pronounced toxicity; he or she will inform the investigators in charge of other patients in the study.

### 18. Regulatory requirements

A form for doctors and dentists exemption certificates must be submitted to the DHSS (form MLA 162) (UK only).

### Appendix 1

| | Onset | Day 1 | Day 2 | Weekly | End of study |
|---|---|---|---|---|---|
| Urine casts | X | | | | X |
| Peak flow | X | X | X | X | X |
| ECG | X | | | After 7 days | X |
| Chest X-ray | X | | | After 7 days | X |
| Clinical history and examination | X | | | X | X |

### Appendix 2

*WHO performance scale*

Scale performance

0 Able to carry out nomal activity without restriction.

1 Restricted in physically strenuous activity but ambulatory and able to carry out light work.

2 Ambulatory and capable of all self-care but unable to carry out any work; up and about more than 50% of waking hours.

3 Capable of only limited self-care; confined to bed or chair more than 50% of waking hours.

4 Completely disabled; cannot carry on any self-care; totally confined to bed or chair.

### Appendix 3

Definitions of toxicity

Toxicity: Always related to a given dose schedule at a given dose level for a given duration of time.

Grade 0 Non-toxic dose: a dose which causes no recognisable effects whatsoever.

Grade 1 Subtoxic dose: a dose which causes consistent changes of haematological or biochemical or other parameters and might thus herald toxicity at the next higher dose level or with prolonged drug administration.

Grade 2 Minimal toxic dose: the smallest dose at which one or more patients show consistent, readily reversible drug toxicity.

Grade 3 The dose which causes moderate reversible toxicity in the majority of patients.

Grade 4 Maximally tolerated dose: the highest tolerable, safe dosage.

### Appendix 4

Tumour masses are counted and measured at regular intervals. Measurable tumour masses are defined as the product of the longest x the widest perpendicular diameter. The third dimension can be added, where possible. Measurability is arbitrarily defined as reproducibility of simultaneous measurements within 50% by independent observers.

1. Complete tumour regression is defined as complete disappearance of all recognisable tumour masses and/or biochemical changes directly related to the tumour.
2. Complete remission is defined as complete tumour regression (1) and complete disappearance of indirect (host mediated) symptoms, signs, haematological and biochemical parameters.
3. Partial regression is defined as >50% decrease of one or more tumour lesions in the absence of progression or occurrence of new lesions elsewhere.
4. Improvement applies only to well-outlined palpable lesions, and is defined as >25 to <50% regression in the absence of progression or occurrence of new lesions elsewhere.
5. Stable disease: changes smaller than those outlined under (3), (4) and (6).
6. Progressive disease: occurrence of any new lesion or increase of well outlined new lesion by 33%.